# Supplementary material for: Application of statistical design to evaluate critical process parameters and optimize formulation technique of polymeric nanoparticles
Source: R Soc Open Sci. 2019 Jul 24;6(7):190896. doi: 10.1098/rsos.190896 (PMC6689589; doi:10.1098/rsos.190896)
Supplement: Supplementary Figures and Tables [file rsos190896supp1.docx]

**Royal Society Open Science**

**Electronic Supplementary Material for**

Application of statistical design to evaluate critical process parameters and optimize formulation technique of polymeric nanoparticles.

Pradipta Sarkar^1^*, Saswati Bhattacharya^2^, Prof. (Dr.) Tapan Kumar Pal^1^

^1^Bioequivalence Study Centre, Department of Pharmaceutical Technology, Jadavpur University, Kolkata, India.

^2^Department of Pharmaceutical Technology, Jadavpur University, Kolkata, India.

**Supplementary Content:**

| **Table S1** | : | Experimental domain and variables of Plackett-Burman design |
| --- | --- | --- |
| **Table S2** | : | Observed responses in Plackett-Burman design. |
| **Figure S1** | : | Main effect plot of factors for the yields of Particle size. |
| **Figure S2** | : | Main effect plot of factors for the yields of Encapsulation efficiency (%EE). |
| **Figure S3** | : | Main effect plot of factors for the yields of Zeta potential. |
| **Figure S4** | : | Predicted value versus Actual value of (A) Particle size, (B) Encapsulation  efficiency (% EE) and (C) Zeta potential in Box–Behnken design. |
| **Figure S5** | : | Overlay plot of Particle size, Encapsulation efficiency (% EE) and Zeta potential in respect of (A) PLGA cont: PVA%, (B) Homogenization speed: PLGA cont., (C) Homogenization speed: PVA% in Box–Behnken design. |

**Supplementary Table S1**:  Experimental domain and variables of Plackett-Burman design.

| **Factor** | **Factor significance** | **Coded variables levels** | |
| --- | --- | --- | --- |
|  |  | **Level (−1)** | **Level (+1)** |
| A | Poly (lactic-co-glycolic acid) (PLGA) content (mg). | 100 | 200 |
| B | Poly(vinyl alcohol) (PVA)Percentage | 0.5 | 5 |
| C | Homogenization Speed (RPM) | 10000 | 30000 |
| D | Homogenization duration (min) | 5 | 10 |
| E | PLGA molecular weight (Mw) | 38000-54000 | 190000-240000 |
| F | PVA molecular weight (Mw) | 31000-50000 | 85000-124000 |
| G | PLGA Terminal Group | Ester terminated | Acid terminated |
| H | PLGA: Drug ratio | 1:1 | 2:1 |

**Supplementary Table S2**:  Observed responses in Plackett-Burman design.

| Run | A | B | C | D | E | F | G | H | Particle Size (nm) | Encapsulation efficiency (%) | Zeta Potential |
| --- | --- | --- | --- | --- | --- | --- | --- | --- | --- | --- | --- |
| 1 | 1 | 1 | -1 | 1 | 1 | 1 | -1 | -1 | 348.5 | 73 | -2.4 |
| 2 | 1 | -1 | 1 | 1 | -1 | 1 | 1 | 1 | 246.3 | 71 | -11.7 |
| 3 | -1 | -1 | -1 | -1 | -1 | -1 | -1 | -1 | 262.4 | 61 | -4.3 |
| 4 | 1 | 1 | -1 | -1 | -1 | 1 | -1 | 1 | 341.8 | 82 | -3.6 |
| 5 | 1 | -1 | -1 | -1 | 1 | -1 | 1 | 1 | 314.6 | 75.7 | -9.3 |
| 6 | 1 | 1 | 1 | -1 | -1 | -1 | 1 | -1 | 239.4 | 83.2 | -10.6 |
| 7 | -1 | 1 | -1 | 1 | 1 | -1 | 1 | 1 | 263.6 | 71.8 | -8.2 |
| 8 | -1 | 1 | 1 | -1 | 1 | 1 | 1 | -1 | 252.8 | 68.1 | -9.8 |
| 9 | -1 | -1 | -1 | 1 | -1 | 1 | 1 | -1 | 327 | 61.5 | -10.3 |
| 10 | 1 | -1 | 1 | 1 | 1 | -1 | -1 | -1 | 218.5 | 75.3 | -4.9 |
| 11 | -1 | 1 | 1 | 1 | -1 | -1 | -1 | 1 | 237.9 | 67.2 | -3.1 |
| 12 | -1 | -1 | 1 | -1 | 1 | 1 | -1 | 1 | 264.7 | 64.2 | -2.3 |


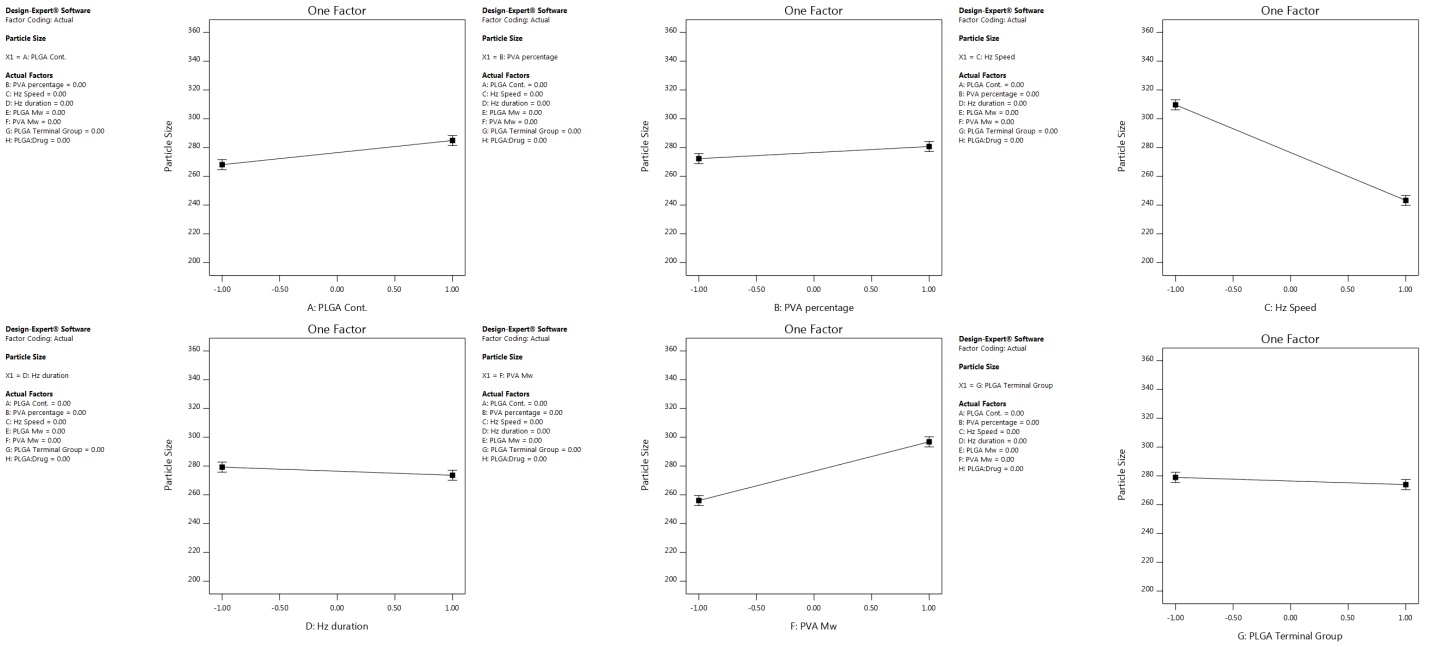


**Supplementary Fig. S1**: Main effect plot of factors for the yields of Particle size.


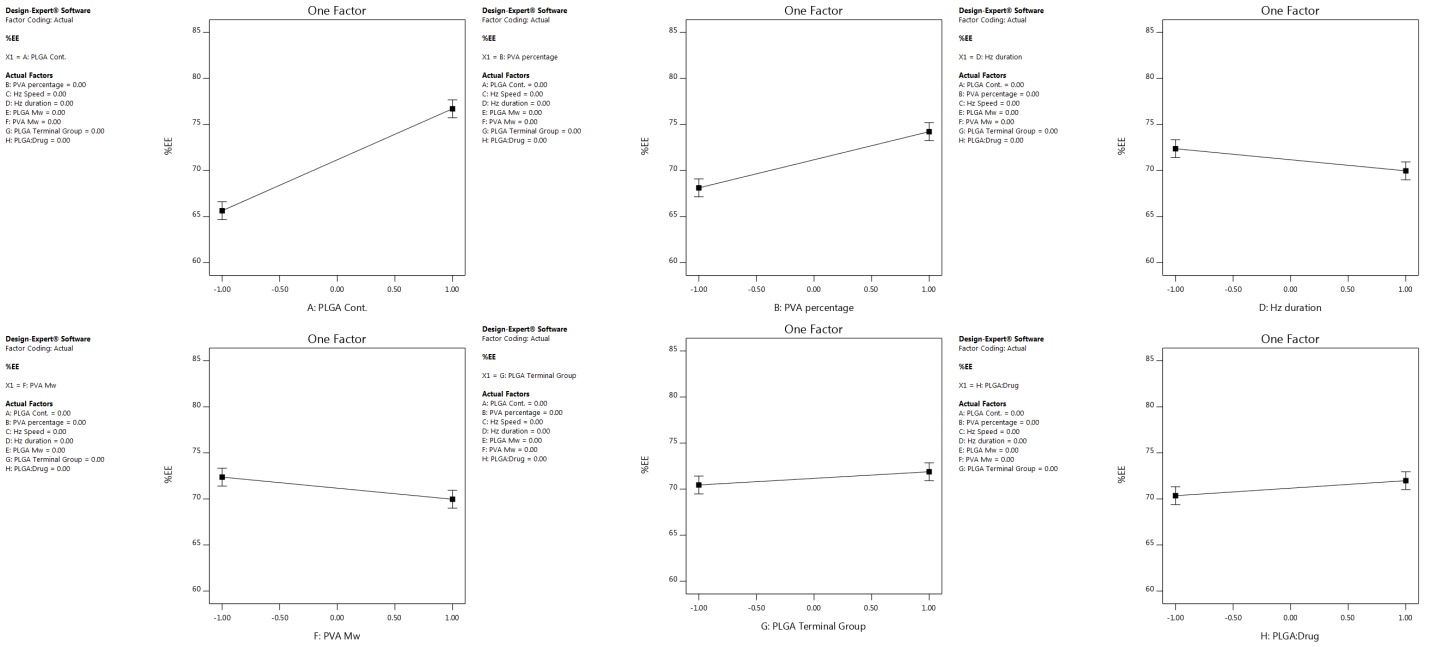


**Supplementary Fig. S2**: Main effect plot of factors for the yields of Encapsulation efficiency (%EE).


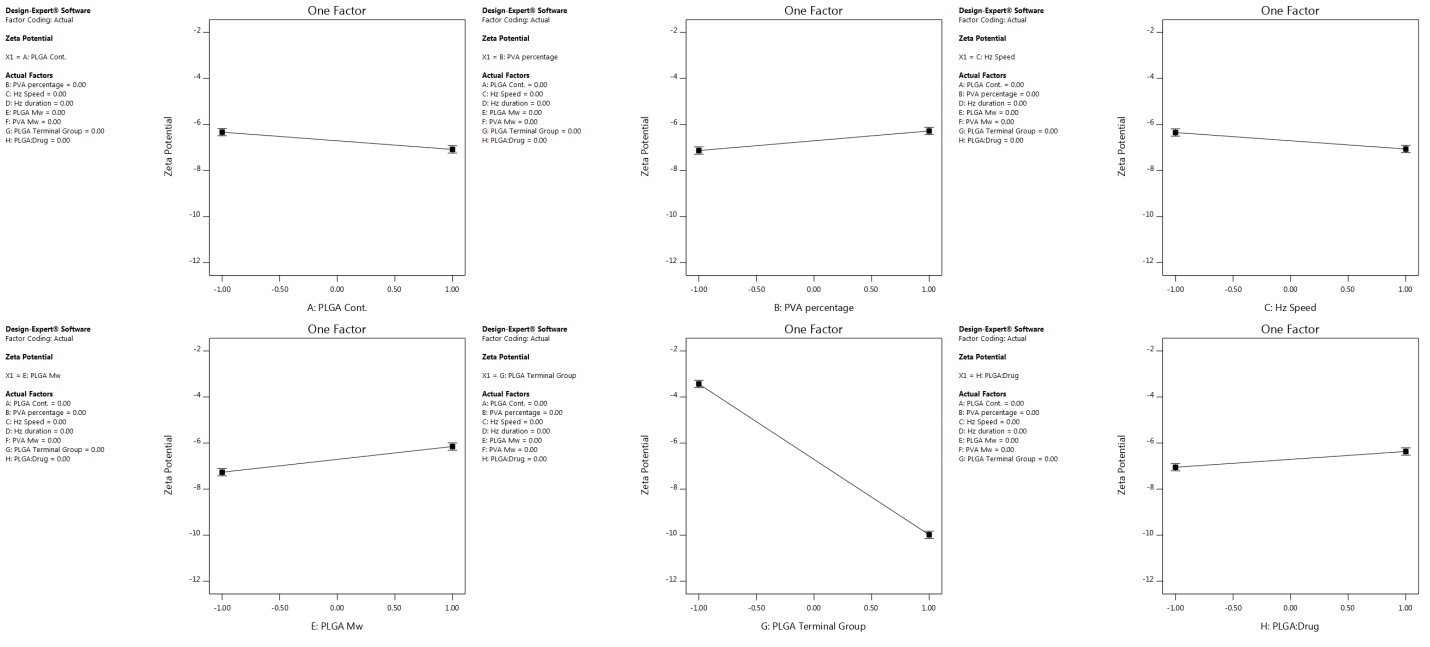


**Supplementary Fig. S3**: Main effect plot of factors for the yields of Zeta potential.


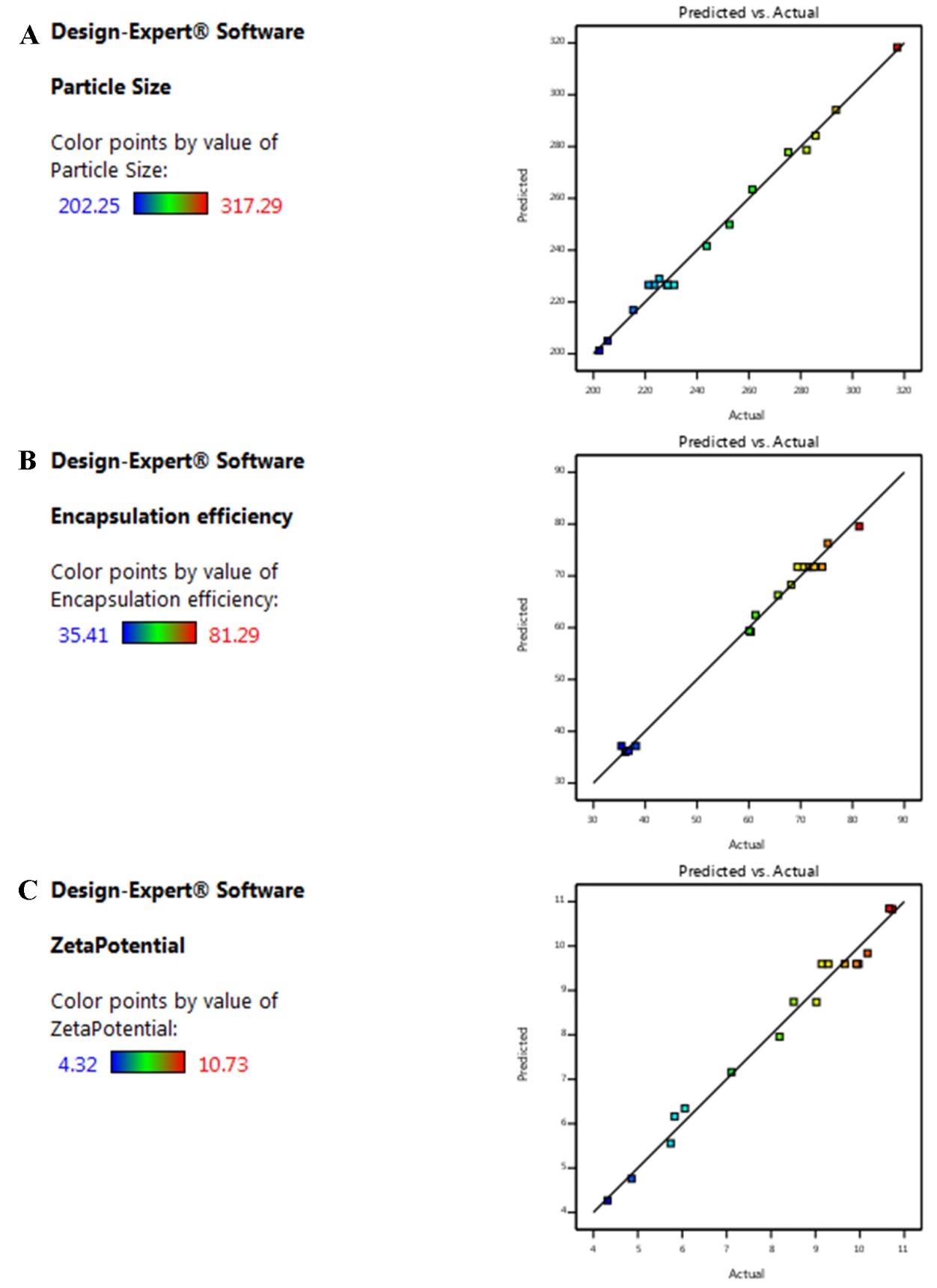


**Supplementary Fig. S4:** Predicted value versus Actual value of (A) Particle size, (B) Encapsulation efficiency (%EE) and (C) Zeta potential in Box–Behnken design.


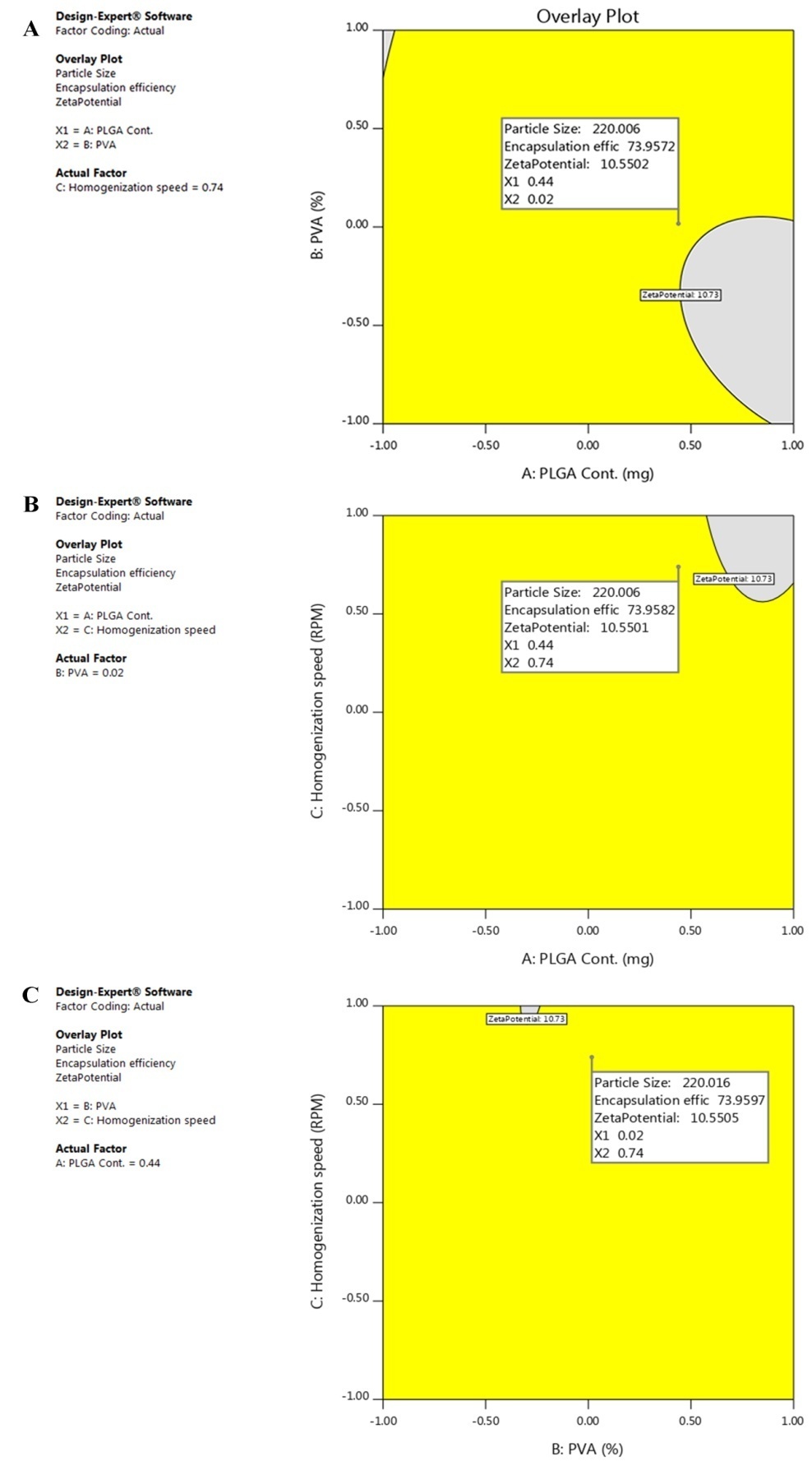


**Supplementary Fig. S5:** Overlay plot of Particle size, Encapsulation efficiency (%EE) and Zeta potential in respect of (A) PLGA cont: PVA%, (B) Homogenization speed: PLGA cont., (C) Homogenization speed: PVA% in Box–Behnken design.
